# Mitigating violence against women and young girls during COVID-19 induced lockdown in Nepal: a wake-up call

**DOI:** 10.1186/s12992-020-00616-w

**Published:** 2020-09-21

**Authors:** Minakshi Dahal, Pratik Khanal, Sajana Maharjan, Bindu Panthi, Sushil Nepal

**Affiliations:** 1 Center for Research on Environment Health and Population Activities (CREHPA), Kathmandu, Nepal; 2 Institute of Medicine, Tribhuvan University, Kathmandu, Nepal; 3 One Heart Worldwide, Kathmandu, Nepal; 4 Nobel College, Pokhara University, Kathmandu, Nepal

## Abstract

Nepal, a South Asian country, was in nationwide lockdown for nearly three months in 2020 with partial restrictions still in place. Much worryingly, COVID-19 induced restrictions have confined women and young girls in their home, increasing the risk of domestic violence. The available support system to respond to violence against women and girls (VAWG) has also been disrupted during this period. The figures of violence against women, and child sexual abuse are increasingly being reported during the lockdown and thereafter. To mitigate this, a response against VAWG should not be a missing agenda. This commentary focuses on the situation of VAWG during COVID-19 induced restrictions in Nepal and offers a way forward for addressing the issue.

## Background

Humanitarian emergency, post-disaster situations, and unrest are a difficult time for people increasing vulnerability for women and children as these situations are linked to an increase in VAWG [1]. COVID-19 is causing a tremendous impact on the world’s economy, and women and girls are facing higher risk as they are already disadvantaged and vulnerable. The fear and uncertainty caused due to the COVID-19 pandemic have intensified various inequalities against women, resulting in violence. There have been reports of an increase in violence against women around the world since the measures like social isolation and lockdown to contain COVID 19 have been imposed [2]. The UN has also identified gender-based violence (GBV) among one of the areas of impacts of COVID-19 on women, others being economy, health, unpaid care work, and in humanitarian and fragile settings and on human rights [3]. Nepal, a resource-constrained economy, ranking 101 out of 153 countries in global gender gap [4], cannot be outside this debate. We here discuss the gender impacts of COVID-19 restrictions in Nepalese women and young girls with a focus on violence, its consequences and way forward.

## Main text

### Confinement and conflict in the home

COVID 19 has forced governments across the world to implement measures to restrict public movement. The situation of home confinement may exacerbate the existing VAWG due to their increased proximity to the perpetrator. Women and young girls might struggle to seek help in such conditions. The compromised support system further poses an increased risk of worsening the violence directed towards them. A nationwide complete lockdown was imposed in Nepal from March 24 to June 14 after which partial restrictions were in place [5]. A total of 885 complaints of domestic violence were received in 24-h toll-free helpline operated by National Women Commission from April to June 2020. This was over twice the number of complaints received within the same period before lockdown (Dec, 2019- Feb,2020, [6]). The number of victims might be higher as they might not have been able to reach out for help due to their continuous scrutiny from the perpetrator. A review by Peterman A et al. also suggest that women tend to stay with abusive partners due to a number of reasons, including social norms, concern for children, and economic reasons [2].

### Livelihood and income

COVID 19 pandemic has caused widespread job loss resulting in economic strain for people. Financial hardship resulting from the pandemic has affected livelihoods, especially for those working in the informal sector. In Nepal, 62.3% of total employed people are engaged in the informal sector [7], which means they have little or no income security and social protection. Economic insecurity has been linked to poor coping strategies like substance use, taking on debt and engaging in risky behaviors, which may trigger for conflict, argument, and interpersonal violence [2]. VAWG can be an outlet or a coping mechanism for some men who feel a loss of control and failure to fulfill traditional breadwinner role during this situation. The influx of thousands of jobless migrant workers from abroad and within the country poses an extra risk of violence against women and children in Nepal.

### Access to health care

COVID-19 pandemic has resulted in an increased burden on the health care system and first responders in Nepal. Due to channelization of resources and other efforts to contain the virus, the health care system is stretched, which has disrupted services to GBV survivors. Also, fear of infection among the victims of the violence may withhold them to visit a health facility for help resulting in a decrease in demand for services against GBV. A finding supports this dynamic during a recent rapid survey conducted by UNFPA, where low utilization of sexual and reproductive health services was found partially attributed to fear among service seekers [8]. Due to the restrictions in movement, women and young girls find it difficult to arrange transportation to visit health facilities or crisis management centers. Similarly, inadequate support from other family members might also complicate seeking help which further adds trauma among them.

### Closure of schools and child marriage

The COVID-19 pandemic is equally threatening for young girls in the country. School closure as a result of lockdown can increase the risk of girls to witnessing violence at home and facing exploitation, violence, and abuse. As per anecdotal records, there have been 48 complaints of child sexual assaults in the first six weeks of lockdown, which is alarming compared to a total of 211 cases in the last Nepali fiscal year (2018/2019, [9]). In most cases, the perpetrator is a relative or a close person indicating the home is not always a safe place for women and young girls. In addition to the abuses, existing harmful practices like child marriage prevalent in the country that disrupts the wellbeing of young girls may be aggravated due to the pandemic. With increasing poverty as a result of the pandemic, parents are more likely to marry off their daughters soon as a mechanism to reduce the economic burden. The economic fallout, in addition to the disruption in various programs and interventions focused on preventing child marriages, is estimated to result in millions of more child marriages in the future [10]. Nepal is one of the top 20 countries with a high prevalence of child marriage (40%) [11]. Some anecdotal records in the country indicate the increase in the prevalence of child marriage during the period of lockdown. Young girls are reported to be getting married by themselves, which is an alarming situation. It is worrisome to note that parents are not bothered to report or intend to bring their daughters back as the family is already in an economic crisis due to the pandemic [12]. Child marriage also results in an increase in school dropout rate as shreds of evidence show that married girls aged 15–17 years are ten times likely to drop out school compared to their unmarried peers in the country [13]. Drop out from schools will prevent young girls to complete their education, which compromises their opportunity to empowerment.

### Impact on mental health

VAWG results in physical as well as psychological effects on victims, which can range from mild anxiety symptoms, worry apprehension, flashbacks and vivid recollections, feeling ashamed to more severe anxiety symptoms, post-traumatic symptoms, and even thoughts of self-harm and suicide. Lack of a support system and ways of escape further worsens the situation. A total of 648 women have been reported to commit suicide during the 83 days of lockdown in the country, which might be partly linked with violence [14]. Women’s stressful situations may further hamper child care in a household, which may impact children’s nutritional as well as psychological wellbeing. Children’s exposure to violence either witnessing it or facing it may disrupt their mental wellbeing.

### Way forward

Regarding the increased vulnerability posed by the COVID 19 towards women and young girls, prevention and management of the VAWG should be included as an essential service in the COVID-19 response plan. This is because the gender impacts of COVID-19 on women and men are different [15]. Health care workers and other frontline workers should be oriented and prepared to address VAWG in this setting. The support shelters should be available, and its access to women must be ensured. These services should be prioritized as essential services without disruption in its operation. Public awareness on various forms of violence, including child marriage and its effects and ways to seek help should be done through mass media mobilization. There should be widespread dissemination of digital platforms for seeking help. There should be strict implementation of the legislation to penalize the perpetrators. Community groups like the mother’s group should be oriented and mobilized during this period to check-in women and children at risk of violence and act as a support system to guide them accordingly. Various social safety nets like paid leave, unemployment insurance, direct cash, or food payments to the poor should be introduced to overcome economic burden, which might reduce violence during the pandemic. Nepal can further benefit from its devolved federal governance by encouraging local governments to prevent, monitor, and manage VAWG. Nevertheless, interventions for the prevention of VAWG should focus on addressing the root causes of the violence shaped by structural, political, social, and economic and policy factors for reducing the gender gap and preventing longer-term impacts of violence.

## Conclusion

In summary, COVID-19 gives a unique environment for countries, including Nepal, to not only deal with the epidemiological aspects of the disease but also with the social elements such as violence, the latter usually the overlooked aspect within the political and health system domain. However, continual negligence in protecting women and girls from violence would mean a setback to the country’s socio-economic potential. This is a wake-up call to the Government of Nepal and all concerned stakeholders and individuals demanding attention and actions for the prevention of VAWG, and the sooner, the better.

## Data Availability

Not applicable.
